# Cytochrome P450 Enzymes Involved in Metoprolol Metabolism and Use of Metoprolol as a CYP2D6 Phenotyping Probe Drug

**DOI:** 10.3389/fphar.2018.00774

**Published:** 2018-07-24

**Authors:** Benjamin Berger, Fabio Bachmann, Urs Duthaler, Stephan Krähenbühl, Manuel Haschke

**Affiliations:** ^1^Division of Clinical Pharmacology and Toxicology, University Hospital Basel, Basel, Switzerland; ^2^Department of Biomedicine, University of Basel, Basel, Switzerland; ^3^Swiss Center for Applied Human Toxicology (SCAHT), Basel, Switzerland; ^4^Clinical Pharmacology and Toxicology, Department of General Internal Medicine, Inselspital, University Hospital Bern, Bern, Switzerland; ^5^Institute of Pharmacology, University of Bern, Bern, Switzerland

**Keywords:** CYP2D6, metoprolol, α-OH-metoprolol, phenotyping, CYP induction

## Abstract

Metoprolol is used for phenotyping of cytochrome P450 (CYP) 2D6, a CYP isoform considered not to be inducible by inducers of the CYP2C, CYP2B, and CYP3A families such as rifampicin. While assessing CYP2D6 activity under basal conditions and after pre-treatment with rifampicin *in vivo*, we surprisingly observed a drop in the metoprolol/α-OH-metoprolol clearance ratio, suggesting CYP2D6 induction. To study this problem, we performed *in vitro* investigations using HepaRG cells and primary human hepatocytes (before and after treatment with 20 μM rifampicin), human liver microsomes, and CYP3A4-overexpressing supersomes. While mRNA expression levels of CYP3A4 showed a 15- to 30-fold increase in both cell models, mRNA of CYP2D6 was not affected by rifampicin. 1′-OH-midazolam formation (reflecting CYP3A4 activity) increased by a factor of 5–8 in both cell models, while the formation of α-OH-metoprolol increased by a factor of 6 in HepaRG cells and of 1.4 in primary human hepatocytes. Inhibition studies using human liver microsomes showed that CYP3A4, 2B6, and 2C9 together contributed 19.0 ± 2.6% (mean ± 95%CI) to O-demethylation, 4.0 ± 0.7% to α-hydroxylation, and 7.6 ± 1.7% to N-dealkylation of metoprolol. In supersomes overexpressing CYP3A4, metoprolol was α-hydroxylated in a reaction inhibited by the CYP3A4-specific inhibitor ketoconazole, but not by the CYP2D6-specific inhibitor quinidine. We conclude that metoprolol is not exclusively metabolized by CYP2D6. CYP3A4, 2B6, and 2C9, which are inducible by rifampicin, contribute to α-hydroxylation, O-demethylation, and N-dealkylation of metoprolol. This contribution is larger after CYP induction by rifampicin but is too small to compromise the usability of metoprolol α-hydroxylation for CYP2D6 phenotyping.

## Introduction

**FIGURE 1:**
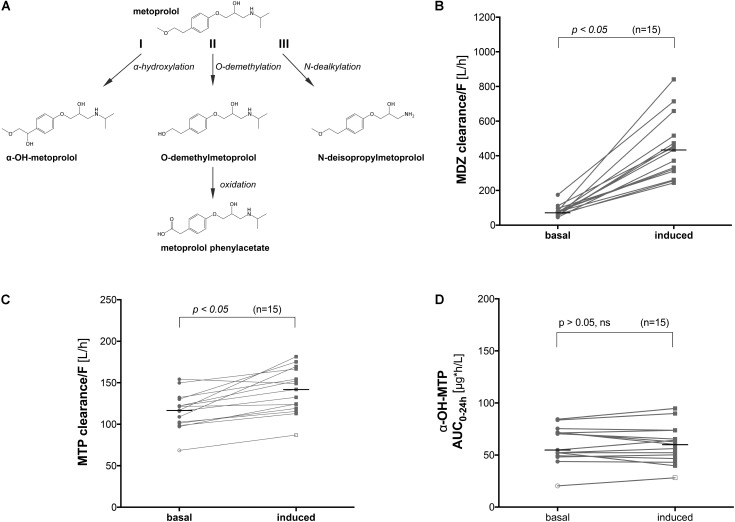
Metabolic pathways of metoprolol and influence of CYP induction on the clearance of metoprolol and midazolam *in vivo*. **(A)** α-Hydroxylation (I), O-demethylation (II), and N-dealkylation (III) represent the 3 principle pathways of metoprolol metabolism, **(B)** midazolam clearance before and after treatment with rifampicin (600 mg rifampicin per day for 7 days), **(C)** metoprolol clearance before and after treatment with rifampicin (600 mg rifampicin per day for 7 days), and **(D)** α-OH-metoprolol exposure before and after treatment with rifampicin (600 mg rifampicin per day for 7 days). MDZ: midazolam, MTP: metoprolol.

Metoprolol is a cardioselective beta-blocker that is used mainly in the treatment of arterial hypertension (Hansson et al., 1999), heart failure (MERIT-HF Study Group, 1999), and myocardial infarction (Chen et al., 2005). Intestinal absorption of metoprolol is rapid and almost complete; however, due to an extensive first pass metabolism (Regardh and Johnsson, 1980), the bioavailability of metoprolol is only approximately 50%. The half-life is in the range of 3–4 h in young adults and between 7–9 h in elderly patients (Rigby et al., 1985). Metoprolol is heavily biotransformed with less than 5% of an oral dose being excreted in non-metabolized form by the kidneys (Regardh and Johnsson, 1980; McGourty et al., 1985; Rigby et al., 1985; Johansson et al., 2007). Approximately 70% of orally administered metoprolol is metabolized by CYP2D6 (Johnson and Burlew, 1996). Major oxidative pathways are O-demethylation to O-demethylmetoprolol and its further oxidation to the corresponding metoprolol phenylacetate (65% of the oral dose recovered in urine); N-dealkylation to N-deisopropylmetoprolol (10%), which may be transaminated and the resulting aldehyde oxidized to the corresponding acid; and α-hydroxylation to α-OH-metoprolol (10%) (Borg et al., 1975) (**Figure 1A**).

Whereas the carboxylic acid metabolites have no pharmacological activity, the α-OH-metabolite and O-demethylmetoprolol are active, but only contribute approximately 10% of the total beta-blocking activity of metoprolol (Regardh et al., 1974). Importantly, in contrast to the formation of the carboxylic acid metabolites, the α-hydroxylation of metoprolol only involves one enzymatic step, which is considered to be mediated exclusively by CYP2D6 (McGourty et al., 1985; Otton et al., 1988). Furthermore, the metabolism of metoprolol is stereoselective; in extensive CYP2D6 metabolizers (CYP2D6 EM), R-metoprolol has a 40% higher clearance than S-metoprolol (Blake et al., 2013).

Due to the exclusive nature by which CYP2D6 is responsible for α-hydroxylation of metoprolol, this step in the metabolic pathway of metoprolol has previously been used for the assessment of CYP2D6 activity *in vivo* (Tamminga et al., 2001; Sharma et al., 2004; Frank et al., 2007; Donzelli et al., 2014; Derungs et al., 2016) and *in vitro* (Birkett et al., 1993). We have recently published a clinical study in healthy volunteers investigating the effect of CYP inhibitors and inducers on the Basel phenotyping cocktail, which contains six low-dosed commercially available drugs (caffeine, efavirenz, losartan, omeprazole, metoprolol, and midazolam) (Derungs et al., 2016). After CYP induction with rifampicin, we not only observed a change in the phenotyping metric for CYP1A2, CYP2B6, CYP2C9, CYP2C19, and CYP3A4, but, albeit small, also for CYP2D6. CYP2D6 activity was evaluated through use of the metabolic ratio of metoprolol and its α-OH-metabolite, as well as the corresponding AUC_0–24__h_ ratio. As CYP2D6 is considered to be non-inducible (Eichelbaum et al., 1986; Rae et al., 2001; Madan et al., 2003; Wenk et al., 2004; Glaeser et al., 2005; Gerets et al., 2012), this result was surprising and difficult to interpret.

Since it has been shown in a clinical study that the metabolism of metoprolol cannot be completely inhibited by quinidine (Johnson and Burlew, 1996), an efficient and specific CYP2D6 inhibitor (Hutzler et al., 2003; Ai et al., 2009), it is likely that apart from CYP2D6, other CYP isoforms are involved in the oxidative degradation of metoprolol, possibly also in its α-hydroxylation. Taking into account the results of our *in vivo* study (Derungs et al., 2016), we predicted that these additional CYPs had to be inducible by rifampicin. In order to solve these questions, we decided to investigate metoprolol metabolism *in vitro* using two different hepatocyte systems as well as human liver microsomes and supersomes. The data obtained by our *in vitro* investigations confirmed that CYPs other than CYP2D6 are involved in metoprolol metabolism, explaining the decrease in the metoprolol/α-OH-metoprolol ratio after treatment with rifampicin observed *in vivo*.

## Materials and Methods

### Chemicals and Reagents

Metoprolol, α-OH-metoprolol, O-demethylmetoprolol, metoprolol phenylacetate, and metoprolol-d7 were purchased from TRC (Toronto, Canada). 1′-OH-midazolam and midazolam-d6 were acquired from Lipomed (Arlesheim, Switzerland), while rifampicin, ketoconazole, quinidine, and β-glucuronidase (type HP-2 from *Helix pomatia*) were obtained from Sigma-Aldrich (Sigma-Aldrich Chemie GmbH, Buchs, Switzerland). Midazolam was provided by Roche (Hoffmann-La Roche, Basel, Switzerland). Formic acid, HPLC grade methanol, and HPLC grade water were purchased from Merck (Darmstadt, Germany). The media used were purchased from GIBCO (Lucerne, Switzerland).

Stock solutions, calibration spiking solutions, and quality controls were prepared in DMSO. Calibration standards and quality controls were prepared by enriching the respective medium with the corresponding spiking solutions. Internal standard solutions containing the deuterated cocktail probe drugs were prepared in methanol.

### Human Liver Microsomes and Cell Culture

Pooled human liver microsomes (HLM; Lot #38289), and recombinant human CYP3A4 supersomes (rhCYP3A4 + P450 reductase + cytochrome b_5_, Lot #4070007), and NADPH regenerating solutions A and B (*A*, containing NADP^+^, glucose-6-phosphate, and MgCl_2_; *B*, containing glucose-6-phosphate dehydrogenase) were purchased from Corning Life science (Woburn, MA, United States). Microsomes and supersomes were stored at −80°C until used.

HepaRG cells were purchased from Biopredic International (Rennes, France) as undifferentiated cryopreserved cells with the associated medium. Freshly split HepaRG cells were seeded at 9000 cells/well in 96-well plates (or 0.05 × 10^6^ cells/well in 24-well plates) and treated over the course of the next 4 weeks as previously described (Aninat et al., 2006).

Primary cryopreserved human hepatocytes (Life Technologies, Lot #Hu8119 and Lot #Hu8124) were plated at 0.05 × 10^6^ cells/well in collagen type-1 precoated 96-well plates (or 0.25 × 10^6^ cells/well in 24-well plates) in William’s E medium supplemented with 10% FCS (v/v), 1% L-glutamine 200 mM (v/v), 1% Pen Strep (v/v), 0.1% dexamethasone 100 μM (v/v), and 0.1% insulin 100 μM (v/v), and then incubated at 37°C in the presence of 5% CO_2_ and 95% humid air.

### *In Vivo* Assessment of CYP Induction

The *in vivo* characterization of the Basel phenotyping cocktail has been described in detail in prior publications (Donzelli et al., 2014; Derungs et al., 2016). The data presented here origin from one of these studies published previously (Derungs et al., 2016). The study had been approved by the local ethics committee (Ethikkommission Basel) and the national regulatory authorities (Swiss Agency for Therapeutic Products, Swissmedic) and has been conducted in accordance with the ethical standards of the Declaration of Helsinki. It was a single-center, randomized, two-way crossover study ^1^ (ID: NCT01386593) that was conducted at the Phase I Research Unit, University Hospital Basel, Switzerland. In this study, CYP induction had been achieved by treating 15 healthy volunteers with 600 mg rifampicin per day for 7 days. Subjects ingested 12.5 mg metoprolol and 2 mg midazolam (and other CYP substrates) before and after CYP induction by rifampicin. Plasma samples were obtained and analyzed as described previously (Derungs et al., 2016). We determined the AUC using the trapezoidal rule and apparent clearance (Cl/F) by dividing the oral dose administered for both metoprolol and midazolam with the respective AUCs. We used the ratio between the induced and the basal state of the Cl/F of midazolam as a marker of CYP3A4 induction.

### Quantification of Gene Expression

HepaRG cells and primary cryopreserved human hepatocytes were seeded in 24-well plates and treated for 48 h with rifampicin 20 μM. A total of 350 μL of RLT buffer (Qiagen, Hombrechtikon, Switzerland) was used to lyse the respective liver cell models, after which the lysate was transferred to Qiashredder columns and centrifuged for 2 min at 13,000 rpm. From the eluate, total RNA was extracted and purified according to the manufacturer’s instructions (Qiagen, RNeasy mini extraction kit). The concentration of the extracted RNA was measured spectrophotometrically at 260 nm on a NanoDrop 2000 (Thermo Fisher Scientific, Wohlen, Switzerland). cDNA was reverse transcribed from the isolated RNA using the Qiagen omniscript system. For quantitative rt-PCR, 10 ng cDNA was used. Forward and reverse primers for all CYPs tested, and endogenous references, Hypoxanthine phosphoribosyltransferase 1 (HPRT1) and GAPDH, were purchased from Microsynth (Balgach, Switzerland; listed in **Table 1**). rt-PCR was performed using SYBR green (Roche Diagnostics, Rotkreuz, Switzerland) on an ABI PRISM 7700 sequence detector (PE Biosystems, Rotkreuz, Switzerland). Quantification of mRNA expression levels was performed using the comparative-threshold cycle method (Livak and Schmittgen, 2001).

**Table 1:** Gene-specific primers for rt-PCR.

Target Gene	Organism	Primer Sequence (5′ → 3′)		Length (bp)
CYP2D6	human	Fw	TGTGCCCATCACCCAGAT	18
		Rev	AAGGTGGAGACGGAGAAGC	19
CYP3A4	human	Fw	TACACAAAAGCACCGAGTGG	20
		Rev	TGCAGTTTCTGCTGGACATC	20
HPRT1	human	Fw	GGTCCTTTTCACCAGCAAGCT	21
		Rev	TGACACTGGCAAAACAATGCA	21
GAPDH	human	Fw	AGCCACATCGCTCAGACAC	19
		Rev	GCCCAATACGACCAAATCC	19

### Functional Assessment of CYP Induction

HepaRG cells and primary cryopreserved human hepatocytes were cultured in a 5% CO_2_ and 95% air-humidified atmosphere at 37°C. Induction treatment (rifampicin 20 μM) lasted for 48 h, with the medium being changed every 24 h. After medium change at 24 h, rifampicin (20 μM) was added again to the medium. Rifampicin stock solution was prepared in DMSO and further diluted in the appropriate culture medium, to achieve a final DMSO concentration of 0.1% (v/v). Experimental control culture wells were treated with solvent [DMSO, 0.1% (v/v)] alone. Following treatment with rifampicin, CYP activity was assessed by the addition of fresh medium containing the probe drugs (metoprolol 25 μM or midazolam 5 μM). Probe drugs were dissolved and serially diluted to the required concentrations with DMSO. The final concentration of DMSO during the incubation was 0.2% (v/v). At selected time points (0, 15, 30, 45, 60, 90, 105, and 120 min), the incubation was stopped by the addition of a threefold volume of ice-cold methanol containing the respective internal standards. The bottoms of the wells were scraped using a pipette tip, after which the contents were transferred to an autosampler vial. After vigorous shaking (10 min) and centrifugation (3220 × *g*; 30 min; 10°C), the supernatants were stored at −20°C until analysis by liquid chromatography-tandem mass spectrometry (LC-MS/MS).

To determine the total amount of 1′-OH-midazolam formed, the entire content of the autosampler vials was evaporated using a minivap microplate evaporator (Porvair Sciences Ltd., King’s Lynn, Norfolk, United Kingdom). The analytes were then re-suspended in 45 μl of the respective culture medium, to which 5 μl (500 units) of β-glucuronidase was added. Following a 12-h incubation at 37°C, the reaction was terminated by the addition of methanol, after which the samples were treated in the same manner as above.

### Metabolism of Metoprolol and Midazolam by Human Liver Microsomes and by rhCYP3A4 Supersomes

The reaction mixture (final volume of 500 μL) contained the probe drugs (metoprolol 25 μM or midazolam 5 μM), incubation buffer (0.1 M potassium phosphate buffered saline, pH 7.4), human liver microsomes (175 pmol cytochrome P450/500 μL) or rhCYP3A4 (10 pmol CYP3A4/500 μL) and a NADPH-generating system. For inhibition studies, the reaction mixture was pre-incubated with the specific inhibitors (concentrations indicated in the figures) for 10 min prior to the addition of test compounds and NADPH. The suspensions were incubated at 37°C in a thermomixer (compact 5350, Eppendorf, Hamburg, Germany). Following the incubation in the absence (control) or presence of inhibitors, the reactions were terminated at selected time-points by transferring 50 μL of the incubation mixture into a matrix autosampler vial, to which a threefold volume of ice-cold methanol (containing the internal standards) was added. After vigorous shaking (10 min) and centrifugation (3220 × *g*; 30 min; 10°C) the samples were stored at −20°C until analysis by LC-MS/MS. The formation rate (pmol/min/pmol P450) in the absence and the presence of the inhibitors was calculated from the linear parts of the concentration-time curves and expressed as an absolute value or as a percentage relative to the control values.

### Quantification of Probe Substrate Metabolites

A previously developed LC-MS/MS method (Donzelli et al., 2014) was used to quantify the phase I metabolites of metoprolol and midazolam. Chromatographic separation was performed on a Shimadzu HPLC system (Shimadzu AG, Reinach, Switzerland), coupled to a triple quadrupole tandem mass spectrometer (API4000 or API5500, AB/MDS Sciex, Concord, Canada), operating in positive electrospray ionization mode. The total run time was 2.9 min. Inter-assay accuracy (determined as the % bias) ranged from −8.6 to 8.9 and inter-assay precision (determined as the CV%) was lower than 8.3 for all analytes. The lower limit of quantification (LLOQ) utilizing the API 4000 was 0.25 ng/mL for α-OH-metoprolol and 1′-OH-midazolam, whereas on the API5500 the LLOQ for α-OH-metoprolol, O-demethylmetoprolol, and metoprolol phenylacetate (see **Figure 1A** for chemical structures) was 0.025 ng/mL.

### Data Analysis

The CYP activities were determined as the respective metabolite formation rates corresponding to the slope in the metabolite concentration versus time graphs. Metabolite concentrations were quantified using standard curves of pure compounds as previously described (Donzelli et al., 2014).

For induction experiments, metabolite formation rates were determined as described above, and the fold change versus the basal conditions was calculated as the ratio of the metabolite formation rate in wells exposed to an inducer versus formation rates in control wells.

Experiments were carried out 5 times unless stated otherwise. Means were compared with the two-tailed Student’s *t*-test using GraphPad Prism 6.0 (GraphPad Software, San Diego, CA, United States). Data are presented as mean ± SD unless stated otherwise. Induction levels above twofold with statistically significant differences (*p* < 0.05) were considered relevant. Areas under the curve (AUC), from times 0–24 h after dosing (AUC_0–24__h_), were estimated with non-compartmental methods using Phoenix WinNonlin software (Certara, Princeton, NJ, United States).

## Results

### *In Vivo* Clearance of Metoprolol Before and After Treatment With Rifampicin

We performed a clinical study using 15 healthy volunteers, where we investigated the effect of pre-treatment with rifampicin on the Basel phenotyping cocktail, which contains caffeine, efavirenz, losartan, omeprazole, metoprolol, and midazolam as probe drugs. We have reported the changes in the metabolic ratios induced by rifampicin of the six substrates included in a previous publication (Derungs et al., 2016). As expected, we observed an increase in the clearance of midazolam (**Figure 1B**) in all subjects tested, which is a marker of CYP3A4 activity (Link et al., 2008). Surprisingly, we also observed a 25% increase in the clearance of metoprolol in subjects treated with rifampicin compared to the basal conditions (**Figure 1C**). An increase by 9% (statistically not significant) was also observed in the formation of α-OH-metoprolol after treatment with rifampicin (**Figure 1D**).

The findings regarding metoprolol suggested either an induction of CYP2D6 by rifampicin and/or metabolism of metoprolol by CYPs other than 2D6, which are inducible by rifampicin. To study the induction of the metabolism of metoprolol by rifampicin in more detail, we performed *in vitro* investigations in HepaRG cells and primary human hepatocytes as well as in human liver microsomes and supersomes.

### mRNA Expression of CYP2D6 and CYP3A4, and Midazolam and Metoprolol Metabolism Before and After Rifampicin in HepaRG Cells

**FIGURE 2:**
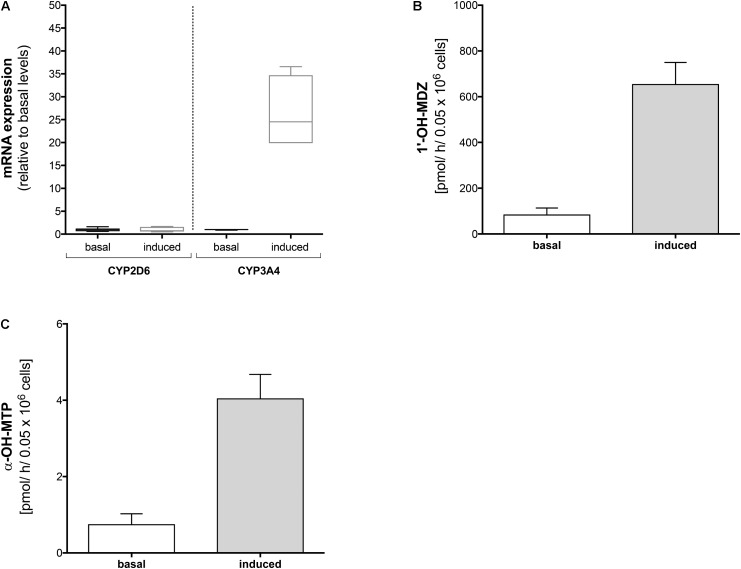
Effect of rifampicin on mRNA expression and metabolism of midazolam and metoprolol in HepaRG cells. HepaRG cells were exposed to 20 μM rifampicin for 48 h. **(A)** mRNA expression of CYP2D6 and CYP3A4 before and after treatment with rifampicin, **(B)** formation of 1′-OH-midazolam before and after treatment with rifampicin, and **(C)** formation of α-OH-metoprolol before and after treatment with rifampicin. Data are presented as mean ± SD. MDZ: midazolam, MTP: metoprolol.

First, we reproduced the *in vivo* findings in HepaRG cells, which contain inducible CYPs (Berger et al., 2016). As shown in **Figure 2**, we investigated the effect of CYP induction by rifampicin on mRNA expression and activity of CYP2D6 and CYP3A4.

**FIGURE 3:**
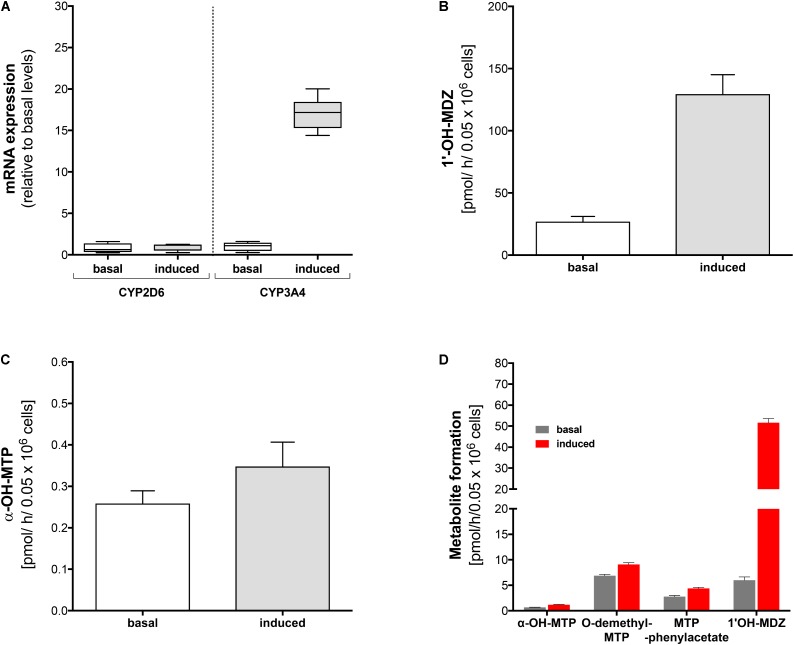
Effect of rifampicin on mRNA expression and metabolism of midazolam and metoprolol in primary human hepatocytes. Primary human hepatocytes were exposed to 20 μM rifampicin for 48 h. **(A)** mRNA expression of CYP2D6 and CYP3A4 before and after treatment with rifampicin, **(B)** formation of 1′-OH-midazolam before and after treatment with rifampicin, **(C)** formation of α-OH-metoprolol before and after treatment with rifampicin, **(D)** formation of 1′-OH-midazolam, α-OH-metoprolol, O-demethyl-metoprolol, and metoprolol phenylacetate before and after treatment with rifampicin. Experiments for **(A–C)** were carried out with lot #Hu8119 and experiments for **(D)** with lot #Hu8124. Data are presented as mean ± SD. MDZ: midazolam, MTP: metoprolol.

In HepaRG cells, mRNA of CYP3A4 (determined as a positive control) increased approximately 30-fold after treatment with rifampicin (20 μM for 48 h) compared to basal conditions (**Figure 3A**). In contrast, mRNA of CYP2D6 was not induced by pre-treatment with rifampicin.

In agreement with the mRNA data, pre-treatment with rifampicin was associated with a significant increase in the formation of 1′-OH-midazolam (**Figure 2B**), reflecting induction of CYP3A4 by rifampicin. In contrast to the results obtained for CYP2D6 mRNA, pre-treatment with rifampicin was also associated with a significant increase in α-OH-metoprolol formation (**Figure 2C**). This result suggested that CYPs other than CYP2D6 contribute to α-hydroxylation of metoprolol and that the contribution becomes larger after CYP induction.

### mRNA Expression of CYP2D6 and CYP3A4, and Midazolam and Metoprolol Metabolism Before and After Rifampicin in 2D Primary Hepatocytes

In order to confirm the results obtained in HepaRG cells, we performed similar experiments in primary human hepatocytes (Lot #Hu8119). Identical to our findings in HepaRG cells, pre-treatment with rifampicin was associated with a 16-fold increase in CYP3A4 mRNA expression, whereas no increase in mRNA expression levels could be observed for CYP2D6 (**Figure 3A**).

Pre-treatment with rifampicin led to a fivefold increase in production of 1′-OH-midazolam (**Figure 3B**). Similar to the findings in HepaRG cells, rifampicin increased α-hydroxylation of metoprolol also in primary human hepatocytes (**Figure 3C**), but to a lesser extent than in HepaRG cells. These findings confirmed the results obtained with HepaRG cells and excluded at the same time an artifact due to modified regulation of CYP expression in the HepaRG hepatoma cell line.

Using a different batch of primary cryopreserved human hepatocytes (Lot #Hu8124) we could confirm the results regarding the formation of 1′-OH-midazolam and α-hydroxylation of metoprolol (**Figure 3D**). Furthermore, we also investigated the formation of O-demethyl-metoprolol and its oxidation to metoprolol phenylacetate. Similar to the increase in the formation of α-OH-metoprolol, pre-treatment with rifampicin was also associated with an increase in O-demethylation of metoprolol (factor 1.4) and in the conversion of O-demethyl-metoprolol to the corresponding phenylacetate metabolite (factor 1.6).

These experiments not only confirmed the previous results in HepaRG cells and primary human hepatocytes, but also showed that CYPs other than CYP2D6 contribute to O-demethylation of metoprolol, the most important metabolic pathway of this drug (Borg et al., 1975; Regardh and Johnsson, 1980).

### CYPs Involved in the Metabolism of Metoprolol in Human Liver Microsomes

**FIGURE 4:**
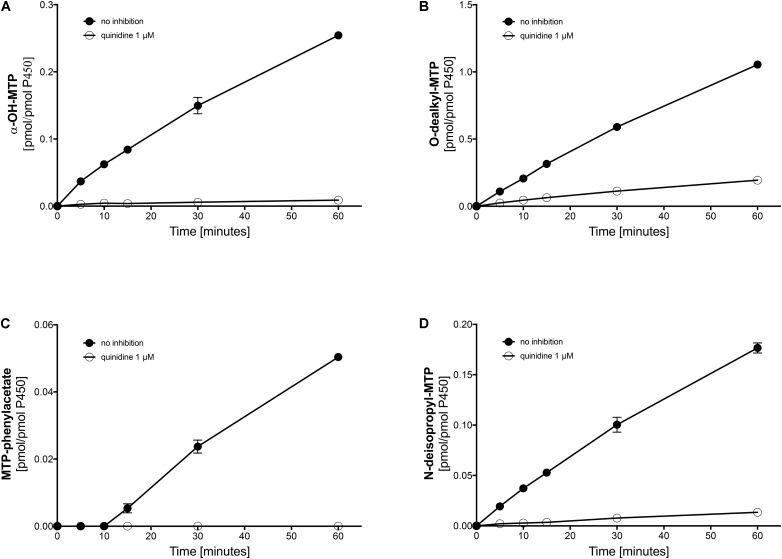
Effect of quinidine on the metabolism of metoprolol by human liver microsomes. The quinidine concentration was 1 μM. **(A)** formation of α-OH-metoprolol, **(B)** formation of O-demethyl-metoprolol, **(C)** formation of metoprolol phenylacetate, and **(D)** formation of N-deisopropyl-metoprolol. Data are analyzed in **Table 2**. Data are presented as mean ± SD. MTP: metoprolol.

Next, we investigated the metabolic pathways of metoprolol in human liver microsomes in the presence of different inhibitors. In the presence of quinidine, a specific and strong inhibitor of CYP2D6 (Hutzler et al., 2003; Ai et al., 2009), α-hydroxylation, O-demethylation, and N-dealkylation of metoprolol was not blocked completely (**Figures 4A–C** and **Table 2**), again suggesting the involvement of CYPs other than CYP2D6 in metoprolol metabolism. In comparison, the formation of the phenylacetate metabolite from O-demethylated metoprolol had a very low activity and was blocked completely by quinidine (**Figure 4D**). These findings were in agreement with the *in vitro* study of Otton et al. (1988) and the *in vivo* studies of Lennard et al. (1982) and of McGourty et al. (1985), showing residual metabolism of metoprolol in the absence of functional CYP2D6.

**Table 2:** Metoprolol metabolism by human liver microsomes in the absence (w/o) and presence of inhibitors.

Metabolite formed	w/o inhibitor fmol/min/pmol CYP	with inhibitor fmol/min/pmol CYP	% residual activity (quinidine) or % inhibition (ketoconazole, ticlopidine, sulfaphenazole, (+)-N-3-benzylnirvanol)
**Quinidine 1 μM (CYP2D6)**			
O-demethyl-MTP	17.5 ± 0.3	3.32 ± 0.40	19.0 ± 2.1 (2.6)
α-OH-MTP	4.11 ± 0.06	0.16 ± 0.06	4.0 ± 0.6 (0.70)
MTP-phenylacetate	0.91 ± 0.0.05	0	0
N-dealkyl-MTP	2.91 ± 0.06	0.22 ± 0.05	7.6 ± 1.7 (2.1)
**Ketoconazole 1 μM (CYP3A4)**			
O-demethyl-MTP	26.6 ± 0.0.6	22.0 ± 0.0.8	17.5 ± 3.4 (4.3)
α-OH-MTP	6.86 ± 0.0.23	5.71 ± 0.29	16.8 ± 2.4 (3.0)
N-dealkyl-MTP	4.86 ± 0.29	4.38 ± 0.23	9.9 ± 1.7 (2.1)
**Ticlopidine 1 μM (CYP2B6)**			
O-demethyl-MTP	30.3 ± 3.3	24.2 ± 0.34	20.2 ± 8.0 (9.9)
α-OH-MTP	7.43 ± 1.14	5.71 ± 0.0.29	23.2 ± 11.1 (13.7)
N-dealkyl-MTP	4.97 ± 1.03	3.74 ± 0.23	24.8 ± 12.7 (15.8)
**Sulfaphenazole 10 μM (CYP2C9)**			
O-demethyl-MTP	27.7 ± 0.40	20.3 ± 2.4	26.5 ± 10.4 (13.2)
α-OH-MTP	6.40 ± 0.23	4.67 ± 0.57	27.0 ± 9.8 (12.2)
N-dealkyl-MTP	4.51 ± 0.0.46	3.83 ± 0.74	15.2 ± 14.5 (18.0)
**(+)-N-3-benzylnirvanol 10 μM (CYP2C19)**			
O-demethyl-MTP	25.8 ± 0.6	25.0 ± 1.43	3.1 ± 8.4 (10.4)
α-OH-MTP	6.23 ± 0.23	6.23 ± 0.0.34	0
N-dealkyl-MTP	4.29 ± 0.17	4.29 ± 0.23	0

*Metoprolol metabolites were determined by LC/MS as described in the Section “Materials and Methods.” Activity is given as pmol/min/pmol CYP. Data are given as mean ± SD and 95%CI in parentheses for residual activity. CYP: cytochrome P450 enzyme, MTP: metoprolol.*

Next, we investigated, which additional CYPs are involved in the metabolism of metoprolol. In the presence of ketoconazole, at the concentration used (1 μM) a specific inhibitor of CYP3A4 (Berger et al., 2016), all major metabolic pathways assessed (O-demethylation, α-hydroxylation, and N-dealkylation) were inhibited significantly by 9.9–17.5% (**Table 2** and Supplementary Figure 1). In comparison, the CYP2B6 inhibitor ticlopidine significantly reduced the activities of all metabolic pathways of metoprolol investigated by 20.2–24.8% (**Table 2** and Supplementary Figure 2). Similarly, the CYP2C9 inhibitor sulfaphenazole reduced the activities of all metabolic pathways by 15.2–27.0%, reaching statistical significance for O-demethylation and α-hydroxylation (**Table 2**, Supplementary Figure 3). In contrast, the CYP2C19 inhibitor (+)-N-3-benzylnirvanol did not significantly decrease the activity of metoprolol metabolism (**Table 2**, Supplementary Figure 4).

These results indicated that the major part of metoprolol is metabolized by CYP2D6, but that CYP3A4, CYP2B6 and CYP2C9 contribute significantly to the metabolism of metoprolol. Since CYP3A4, 2B6, and 2C9 are inducible by rifampicin (Berger et al., 2016), the results obtained in microsomes supported our *in vivo* findings as well as the results obtained in HepaRG cells and in primary human hepatocytes.

### α-Hydroxylation of Metoprolol by rhCYP3A4 Supersomes

In order to demonstrate directly that CYP3A4 can α-hydroxylate metoprolol, we performed experiments using recombinant supersomes expressing CYP3A4 (rhCYP3A4). To ensure that these supersomes do not contain CYP2D6 activity, we exposed them to dextromethorphan, a typical CYP2D6 substrate (Frank et al., 2007). The conversion to dextrorphan was not quantifiable, excluding a significant CYP2D6 activity (data not shown).

**FIGURE 5:**
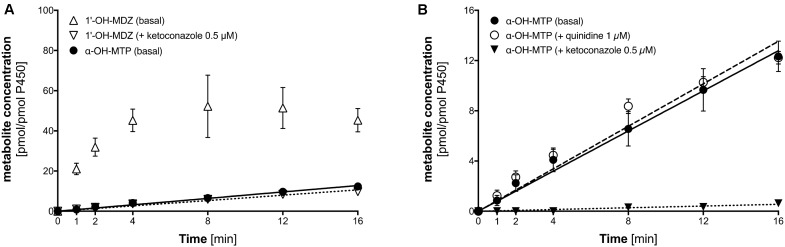
α-Hydroxylation of metoprolol and 1′-hydroxylation of midazolam by CYP3A4 overexpressing supersomes. **(A)** α-Hydroxylation of metoprolol and 1′-hydroxylation of midazolam. 1′-hydroxylation of midazolam was also determined in the presence of 0.5 μM ketoconazole (CYP3A4 inhibitor). **(B)** α-hydroxylation of metoprolol in the absence and in the presence of 1 μM quinidine (CYP2D6 inhibitor) or 0.5 μM ketoconazole (CYP3A4 inhibitor). Data are presented as mean ± SD. MDZ: midazolam, MTP: metoprolol.

As shown in **Figure 5A**, the formation of 1′-OH-midazolam from midazolam by CYP3A4 overexpressing supersomes was rapid, showing non-linearity after 4 min of incubation due to substrate depletion. Midazolam 1′-hydroxylation could almost completely be inhibited by 1 μM ketoconazole, at this concentration a specific CYP3A4 inhibitor (Berger et al., 2016). Crucially, metoprolol was also α-hydroxylated by CYP3A4 supersomes, but at a much slower rate compared to midazolam.

As shown in **Figure 5B**, α-hydroxylation of metoprolol could be inhibited by 1 μM ketoconazole, but not by quinidine, which is a specific CYP2D6 inhibitor (Hutzler et al., 2003; Ai et al., 2009). This proved directly that α-hydroxylation of metoprolol could not only be catalyzed by CYP2D6 but to a small extent also by CYP3A4.

## Discussion

Starting from our *in vivo* observation that treatment with rifampicin was associated with an increase in the clearance of metoprolol, we investigated the metabolic pathways of metoprolol in different metabolic systems *in vitro*. In HepaRG cells and primary human hepatocytes, we could show that not only CYP2D6 contributes to metoprolol metabolism, but also other CYP isoforms which are inducible by rifampicin. Subsequently, we could demonstrate in human liver microsomes that CYP2D6 is the dominant CYP for metoprolol metabolism, but that CYP3A4, 2B6, and 2C9 are also involved in the three major pathways of metoprolol degradation. Finally, we could confirm these findings directly using CYP3A4 overexpressing supersomes, which had no CYP2D6 activity but could perform metoprolol α-hydroxylation.

The *in vivo* investigations showed a 25% increase in metoprolol clearance, but only a small (9%) increase in the AUC of α-OH-metoprolol after CYP induction with rifampicin. This is consistent with previous findings, which also showed a decrease in the AUC of metoprolol after CYP induction (Haglund et al., 1979; Bennett et al., 1982; Derungs et al., 2016). The *in vivo* results could be reproduced in two hepatocyte models, HepaRG cells (a hepatoma cell line) and primary human hepatocytes. The results obtained in these cell systems showed that both α-hydroxylation and O-demethylation of metoprolol were induced by pre-treatment with rifampicin, while CYP2D6 mRNA expression was not affected.

The lacking increase in CYP2D6 mRNA expression by rifampicin observed in the current study agrees with other studies in primary human hepatocytes (Rae et al., 2001; Gerets et al., 2012; Berger et al., 2016). In enterocytes of human subjects treated with 600 mg rifampicin for 10 days, the CYP2D6 protein expression increased by a factor of 1.6 without reaching statistical significance (Glaeser et al., 2005). Post-transcriptional mechanisms increasing CYP2D6 activity cannot be excluded based on the current study, but are an unlikely cause for our observations. In support of this interpretation, sparteine metabolic clearance, an *in vivo* marker of CYP2D6 activity (McGourty et al., 1985), increased by a factor of 1.3 in subjects treated with rifampicin without reaching statistical significance (Eichelbaum et al., 1986). Similarly, in primary human hepatocytes, the activity of dextromethorphan O-demethylation increased by a factor of 1.3 by treatment with rifampicin without reaching statistical significance (Madan et al., 2003). In the same publication, phenobarbital had an almost identical effect on dextromethorphan O-demethylation as rifampicin (Madan et al., 2003). Similarly, hypericum extract did not significantly affect the metabolic ratio of dextrorphan/dextromethorphan, indicating that CYP2D6 is not induced by this treatment (Wenk et al., 2004).

The data of the current and the previous studies mentioned above show that CYP2D6 activity is not increased by the classical inducers of the CYP2B, 2C, and 3A families. The observed increase in the metoprolol metabolic pathways after treatment with rifampicin suggested therefore the contribution of CYPs other than CYP2D6 to metoprolol metabolism. In order to challenge this hypothesis further, we investigated the metabolism of metoprolol in human liver microsomes and in CYP3A4 overexpressing supersomes. The data obtained in microsomes confirmed the contribution of CYP3A4, 2B6, and 2C9 (but not 2C19) to all 3 major metabolic pathways of metoprolol. Finally, using CYP3A4 overexpressing supersomes, we could demonstrate directly that metoprolol can be α-hydroxylated by CYP3A4.

In human liver microsomes, inhibition of CYP2D6 activity with quinidine revealed residual activities amassing to 4% for α-hydroxylation, 8% for N-dealkylation, and 19% for O-demethylation of metoprolol. Considering these values, it has to be taken into account, that the microsomes used in the current study were obtained from donors without CYP induction. Based on the data of the current and previous studies (Haglund et al., 1979; Bennett et al., 1982; Derungs et al., 2016), it can be assumed that the contribution of CYP3A4, CYP2C9, and CYP2B6 becomes larger after CYP induction. In primary human hepatocytes treated with rifampicin, the production of the metoprolol phenylacetate metabolite (the end product of the O-demethylation pathway) and of α-OH-metoprolol increased by 57 and 75%, respectively (**Figure 3D**), suggesting that the contribution of CYPs other than CYP2D6 for metoprolol metabolism can be substantial after CYP induction. The *in vivo* study revealed a 25% decrease in the metoprolol AUC, which is less than expected from the *in vitro* experiments. This difference may be due to a more efficient CYP induction under *in vitro* conditions, depending on the availability of rifampicin. In comparison, the increase in the AUC of α-OH-metoprolol in the *in vivo* study was only 9%, which can be explained by the only small contribution of CYPs other than CYP2D6 to this metabolic step (**Figure 4A**).

The question now arises whether metoprolol can be recommended as a substrate for CYP2D6 phenotyping, taking into account the contribution of CYPs other than CYP2D6 to metoprolol metabolism. The problem could be that CYP2D6 poor metabolizers might not be recognized due to the contribution of CYPs other than CYP2D6, which are not affected by this polymorphism. In the study of McGourty et al. (1985), there was a clear separation in renal excretion of α-OH-metoprolol into debrisoquine EM and PM. Also in the studies of Tamminga et al. (2001) and of Derungs et al. (2016), CYP2D6 efficient and poor metabolizers could clearly be distinguished using α-OH-metoprolol plasma concentrations or the metoprolol/α-OH-metoprolol ratio. A further potential problem could be the distinction between CYP2D6 efficient and poor metabolizers in patients with CYP induction. However, also in this situation, it was possible to correctly classify patients using their respective α-OH-metoprolol plasma concentrations or the metoprolol/α-OH-metoprolol ratio (Derungs et al., 2016). The findings of the current and of our previous study (Derungs et al., 2016) suggest that the plasma α-OH-metoprolol concentration is a better marker of CYP2D6 activity than the ratio of the metoprolol/α-OH-metoprolol concentrations, since the contribution of CYPs different from CYP2D6 is smaller for metoprolol α-hydroxylation than for metoprolol O-demethylation or N-dealkylation. However, as shown in the study of Derungs et al. (2016), a correct classification of CYP2D6 poor metabolizers using the metoprolol/α-OH-metoprolol ratio was possible under basal conditions and also after CYP-induction.

## Conclusion

CYP3A4, 2B6, and 2C9 contribute to metoprolol metabolism, including α-hydroxylation. This contribution explains a more pronounced decrease in the metoprolol and a more pronounced increase in the α-OH-metoprolol plasma concentration after CYP induction by rifampicin compared to basal conditions. However, the contribution of CYPs other than CYP2D6 is low and does not impair the usability of the metoprolol/α-OH-metoprolol metabolic ratio for CYP2D6 phenotyping, even in patients with CYP induction by rifampicin.

## Author Contributions

BB performed the experiments, discussed the results, and wrote parts of the paper. FB performed the experiments, discussed the results, and wrote parts of the paper. UD performed the experiments, discussed the results, and wrote parts of the paper. SK designed the experiments, supervised the work, and wrote the paper. MH designed the experiments, supervised the work, and wrote the paper.

## Conflict of Interest Statement

The authors declare that the research was conducted in the absence of any commercial or financial relationships that could be construed as a potential conflict of interest.

## Footnotes

1 clinicaltrials.gov
