# Supplementary material for: Cytochrome P450 Enzymes Involved in Metoprolol Metabolism and Use of Metoprolol as a CYP2D6 Phenotyping Probe Drug
Source: Front Pharmacol. 2018 Jul 24;9:774. doi: 10.3389/fphar.2018.00774 (PMC6066528; doi:10.3389/fphar.2018.00774)
Supplement: Supplementary file 5 [file Data_Sheet_1.DOCX]

**Legends to supplementary Figures**

Supplementary Figure 1. *Effect of ketoconazole on the metabolism of metoprolol and midazolam by human liver microsomes*. The concentration of the CYP3A4 inhibitor ketoconazole was 1 µM. (A) formation of α-OH-metoprolol, (B) formation of O-demethyl-metoprolol, (C) formation of N-deisopropyl-metoprolol, (D) formation of 1’OH-midazolam (positive control). Data are analyzed in Table 2. Data are presented as mean±SD.

Supplementary Figure 2. *Effect of ticlopidine on the metabolism of metoprolol and efavirenz by human liver microsomes*. The concentration of the CYP2B6 inhibitor ticlopidine was 1 µM. (A) formation of α-OH-metoprolol, (B) formation of O-demethyl-metoprolol, (C) formation of N-deisopropyl-metoprolol, (D) formation of OH-efavirenz (positive control). Data are analyzed in Table 2. Data are presented as mean±SD.

Supplementary Figure 3. *Effect of sulfaphenazole on the metabolism of metoprolol and flurbiprofen by human liver microsomes*. The concentration of the CYP2C9 inhibitor sulfaphenazole was 10 µM. (A) formation of α-OH-metoprolol, (B) formation of O-demethyl-metoprolol, (C) formation of N-deisopropyl-metoprolol, (D) formation of OH-flurbiprofen (positive control). Data are analyzed in Table 2. Data are presented as mean±SD.

Supplementary Figure 4. *Effect of (+)-N-3-benzylnirvanol on the metabolism of metoprolol and omeprazole by human liver microsomes*. The concentration of the CYP2C19 inhibitor (+)-N-3-benzylnirvanol was 10 µM. (A) formation of α-OH-metoprolol, (B) formation of O-demethyl-metoprolol, (C) formation of N-deisopropyl-metoprolol, (D) formation of OH-omeprazole (positive control). Data are analyzed in Table 2. Data are presented as mean±SD.
